# RNA Extraction from Endoscopic Ultrasound-Acquired Tissue of Pancreatic Cancer Is Feasible and Allows Investigation of Molecular Features

**DOI:** 10.3390/cells9122561

**Published:** 2020-11-30

**Authors:** Livia Archibugi, Veronica Ruta, Valentina Panzeri, Miriam Redegalli, Sabrina Gloria Giulia Testoni, Maria Chiara Petrone, Gemma Rossi, Massimo Falconi, Michele Reni, Claudio Doglioni, Claudio Sette, Paolo Giorgio Arcidiacono, Gabriele Capurso

**Affiliations:** 1 Pancreato-Biliary Endoscopy and Endosonography Division, Pancreas Translational & Clinical Research Center, IRCCS San Raffaele Scientific Institute, 20132 Milan, Italy; archibugi.livia@hsr.it (L.A.); testoni.sabrinagloriagiulia@hsr.it (S.G.G.T.); petrone.mariachiara@hsr.it (M.C.P.); rossi.gemma@hsr.it (G.R.); capurso.gabriele@hsr.it (G.C.); 2 Department of Pathology, San Raffaele Scientific Institute IRCCS-Vita Salute San Raffaele University, 20132 Milan, Italy; redegalli.miriam@hsr.it (M.R.); falconi.massimo@hsr.it (M.F.); doglioni.claudio@hsr.it (C.D.); 3 Department of Neuroscience, Section of Human Anatomy, Catholic University of the Sacred Heart, 00168 Rome, Italy; veronica.ruta@outlook.it (V.R.); valentina.panzeri@unicatt.it (V.P.); claudio.sette@unicatt.it (C.S.); 4 Fondazione Policlinico Agostino Gemelli IRCCS, 00168 Rome, Italy; 5 Pathology Department, Pancreas Translational & Clinical Research Center, IRCCS San Raffaele Scientific Institute, 20132 Milan, Italy; 6 Pancreatic Surgery Unit, Pancreas Translational & Clinical Research Center, San Raffaele Scientific Institute IRCCS, 20132 Milan, Italy; 7 Department of Medical Oncology, Pancreas Translational & Clinical Research Center, San Raffaele Scientific Institute IRCCS, 20132 Milan, Italy; reni.michele@hsr.it

**Keywords:** pancreatic cancer, EUS, molecular subtypes, FNA, FNB, RNA, optimization, extraction

## Abstract

Transcriptome analyses allow the distinguishing of pancreatic ductal adenocarcinoma (PDAC) subtypes, exhibiting different prognoses and chemotherapy responses. However, RNA extraction from pancreatic tissue is cumbersome and has been performed mainly from surgical samples, which are representative of < 20% of cases. The majority of PDAC patients undergo endoscopic ultrasound (EUS)-guided tissue acquisition (EUS-TA), but RNA has been rarely extracted from EUS-TA with scanty results. Herein, we aimed to determine the best conditions for RNA extraction and analysis from PDAC EUS-TA samples in order to carry out molecular analyses. PDAC cases underwent diagnostic EUS-TA, with needles being a 25G fine needle aspiration (FNA) in all patients and then either a 20G lateral core-trap fine needle biopsy (FNB) or a 25G Franseen FNB; the conservation methods were either snap freezing, RNALater or Trizol. RNA concentration and quality (RNA integrity index; RIN) were analyzed and a panel of genes was investigated for tissue contamination and markers of molecular subtype and aggressivity through qRT-PCR. Seventy-four samples from 37 patients were collected. The median RNA concentration was significantly higher in Trizol samples (10.33 ng/uL) compared with snap frozen (0.64 ng/uL; *p* < 0.0001) and RNALater (0.19 ng/uL; *p* < 0.0001). The RIN was similar between Trizol (5.15) and snap frozen samples (5.85), while for both methods it was higher compared with RNALater (2.7). Among the needles, no substantial difference was seen in terms of RNA concentration and quality. qRT-PCR analyses revealed that samples from all needles were suitable for the detection of PDAC subtype markers (GATA6 and ZEB1) and splice variants associated with mutational status (GAP17) as well as for the detection of contaminating tissue around PDAC cells. This is the first study that specifically investigates the best methodology for RNA extraction from EUS-TA. A higher amount of good quality RNA is obtainable with conservation in Trizol with a clear superiority of neither FNA nor FNB needles. RNA samples from EUS-TA are suitable for transcriptome analysis including the investigation of molecular subtype and splice variants expression.

## 1. Introduction

Pancreatic ductal adenocarcinoma (PDAC) is predicted to become the second leading cause of cancer-related mortality with a five year survival rate of only 8% [1,2]. The latest guidelines have started to acknowledge the importance of germline testing for relevant mutations such as BRCA1 and 2 for the use of platinum-based therapy followed by the recently approved PARP-inhibitors [3] as also of somatic molecular profiling to guide chemotherapy [4,5]. There are, in fact, data reporting a significantly improved overall survival for PDAC patients receiving matched therapies following molecular profiling of the tumor [6].

In the latest years, transcriptomic signatures have also shown potential for the prediction of prognosis and response to chemotherapy beside mutations [7,8]. In particular, two main molecular subtypes of PDAC have been identified, named classical and basal-like, which are characterized by a different prognosis and response to therapy [7,8,9,10,11]. These subtypes have been identified through RNA sequencing studies performed on PDAC tissue derived mostly from surgical specimens. However, at diagnosis only 20% of PDAC patients are eligible for surgery [12], whereas the majority presents either with a locally advanced or a metastatic disease and is directly addressed to chemotherapic treatments. Therefore, it is currently unknown to what extent the two subtypes identified in resectable PDAC are also representative of the majority of PDAC patients. Among the evaluated biomarkers, GATA6 has recently raised attention and has been proposed in a variety of studies as a marker of the classical PDAC subtype [13,14]. Another interesting marker reported in the literature is ZEB1, a transcription factor regulator of epithelial-to-mesenchymal transition, which is also associated with poor prognosis and resistance to gemcitabine [15,16,17]. Furthermore, a recent paper by Escobar-Hoyos et al. reported how the inclusion of a PolyC exone in a GAP17 splicing event was linked to P53 missense hotspot mutations present in about 40% of PDAC and known to be associated with a more aggressive disease [18].

Endoscopic ultrasound tissue acquisition (EUS-TA), either with fine needle aspiration (FNA) or biopsy (FNB), represents the method of choice for the diagnosis of most PDAC patients, with a complication rate of less than 1% [19]. The evaluation of somatic mutations on DNA samples extracted from tissue acquired through EUS-FNA and FNB has already been performed in a few studies [20,21,22]. By contrast, the extraction and analysis of RNA from pancreatic tumors isolated by EUS has been rarely performed and the few attempts have yielded scanty results [23,24,25,26,27]. With the aim of enacting a personalized medicine approach also for PDAC patients, an effective method to acquire a high quality of RNA usable for transcriptomics is fundamental.

The primary aim of our study was to determine the best method in terms of tissue conservation and needle choice to obtain RNA of a good amount and quality from PDAC samples through EUS-TA. The secondary aim was to investigate the possibility of using EUS-TA derived RNA samples for transcriptome analyses aimed at measuring tissue contamination and at classifying tumor subtypes or aggressiveness.

## 2. Materials and Methods

### 2.1. Study Design and Population

**Figure 1:**
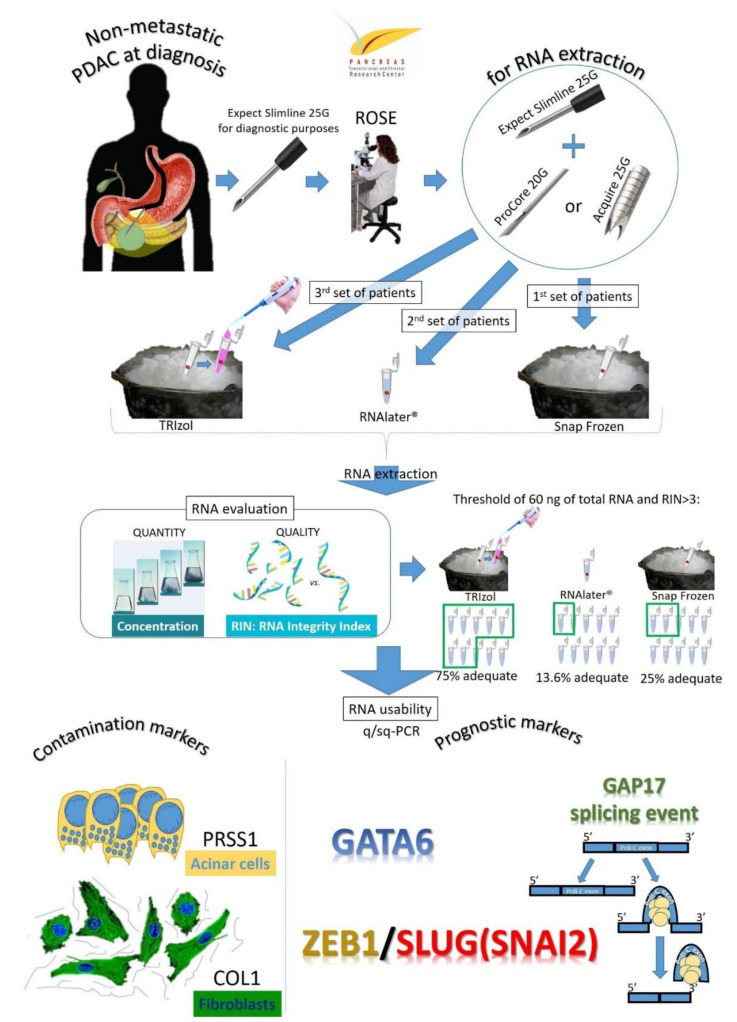
Explanatory representation of the study design.

This was a prospective observational study conducted upon approval of the Internal Review Board (IRB BIOGASTRO/2011 updated on 6 November 2017). Patients with a clinical history and radiologic imaging suggestive of non-metastatic PDAC diagnosis were considered for potential enrollment in the study upon informed consent. Patients were naïve to any treatment before the EUS. Exclusion criteria are reported in Supplementary Figure S1. An explanatory representation of the study design is reported in Figure 1.

### 2.2. Endoscopic Ultrasound Procedure Description and Specimen Processing

EUS procedures were performed under deep sedation with an intravenous infusion of Propofol (Diprivan^®^, Zeneca, Germany) using a Pentax therapeutic linear echoendoscope (EG3870UTK, EG38J10UT) and Hitachi ultrasound platforms (Arietta 850, Arietta V70) by expert endoscopists who perform more than 500 EUS procedures a year. The evaluation of the lesion size and features was performed either from the stomach in the case of body and tail lesions or from the duodenal bulb and the second portion in the case of pancreatic head masses. The EUS-TA was performed from the position that could provide the best stability of the scope, the closest position of the lesion to the probe and the best window for puncture in terms of the absence of blood vessel interposition and evaluated with a color Doppler.

We compared three different needles and for all of them the slow-pull technique was employed. The first EUS-TA was always performed with a 25G FNA needle (Expect Slimline^®^, Boston Scientific, Marlborough, MA, USA) for diagnostic purposes and the sample was expressed onto a clean glass slide with the reinsertion of the stylet. At least two slide smears were prepared in each case, which were fixed in absolute alcohol and stained with hematoxylin and eosin using standard techniques. An onsite cytopathologist would then examine the slides and provide a real-time impression on the adequacy and nature of the lesion and whether the nature of the cells was benign (when the cytologic specimen did not reveal malignancy), suspicious or malignant. The diagnosis was based on pathologic criteria taking into consideration tissue architecture, pleomorphism, hyperchromatic nuclei, high nuclear-to-cytoplasmic ratio and the presence of giant cells. In the case of no adequacy, another pass with the 25G FNA needle was performed and reevaluated. If also the second pass did not retrieve the adequacy, the patient was excluded from this study.

In the case of an adequate sample suspicious for malignancy, the patient would undergo another pass with the 25G FNA needle (Expect Slimline^®^, Boston Scientific) and then either a pass with a 20G needle (ProCore, Cook Medical, Bloomington, IN, USA) or with a 25G needle (Acquire^®^, Boston Scientific). These FNB needles were alternated for each consecutive patient with the slow-pull technique being employed for all cases. Both samples were then expressed in two different vials. The first set of 14 consecutive patients (enrolled between November 2018 and January 2019) had the samples expressed into empty vials positioned in dry ice and then immediately stored at −80 °C. The second set of 11 consecutive patients (enrolled between February 2019 and April 2019) had the samples expressed into a vial containing 600 uL of RNALater (Invitrogen^®^, ThermoFisher, Waltham, MA, USA) at room temperature for at least 1 h and then stored at −80 °C. The third set of 12 consecutive patients (enrolled between May 2019 and October 2019) had the samples expressed into empty vials positioned in dry ice and then immediately taken to the laboratories where 1000 uL of Trizol (Invitrogen^®^, ThermoFisher) was added to the vial under a chemical hood and then stored at −80 °C. A total of nine experimental conditions were therefore set, matching three different needle types with three different conservation methods. No sample size calculation was performed ahead of the study.

### 2.3. RNA Extraction and Analysis

RNA extraction was performed using the RNeasy Plus Mini Kit (Qiagen^®^, Hilden, Germany) according to the manufacturer’s protocol. For homogenization, a hand-held battery operated homogenizer VWR Pellet Mixer (VWR International, Radnor, PA, USA) was employed, working on the sample while on ice until no floating fragment of the tissue was seen in the vial. The final RNA extracted was diluted in 30 uL of RNAse-free water. RNA quantification and evaluation of integrity was performed using a 2100 Bioanalyzer in combination with the RNA 6000 Pico kit (Agilent Technologies, Santa Clara, CA, USA).

### 2.4. Gene Expression and Splicing Assay Analysis

In order to check whether the samples obtained with the above mentioned methods were useful for transcriptome analyses, we planned to employ 20 samples acquired from 10 patients among those characterized by having both FNA and FNB samples with adequate RIN (> 3) and quantity (> 60 ng) to perform real-time quantitative PCR (qPCR) for PRRS1 and COL1 as markers of acinar and stromal contamination, respectively, and GATA6 and ZEB1 (as also SLUG) as markers of classical subtype and epithelial-to-mesenchymal transition (EMT) and semi-quantitative PCR (sqPCR), respectively, for the splicing analysis of ARHGAP17 [18] (see Supplementary Table S1 for primers). A total of 50 ng of RNA was retrotranscribed with M–MLV reverse transcriptase (Promega) and random primers (Roche). The gene expression and splicing patterns were evaluated by semi-quantitative (sqPCR) and qPCR analyses using 2 ng of cDNA template. The percentage of Spliced-In Index (PSI/ψ) was calculated from the densitometric analysis of PCR products as ψ = exon inclusion**/**(exon inclusion + exon skipping) band intensities. qPCR analysis was carried out using a LightCycler 480 SYBR Green I Master (Roche) and a LightCycler^®^ 96 Real-Time PCR System (Roche) system (Applied Biosystems) according to the manufacturer’s instructions. To quantify the relative changes in gene expression, the 2^-ΔΔCq^ method was used and the reactions were normalized on the endogenous control gene L34.

### 2.5. Statistical Analysis

Descriptive statistics were calculated as means and standard deviation (SD) for normally distributed continuous variables, as means and SD and median and interquartile range (IQR) for continuous variables with skewed distribution and as numbers and percentages for categorical variables.

An unpaired T-test was employed for the comparison of normally distributed continuous variables and a Mann–Whitney U test for continuous variables with skewed distribution. One-way analysis of variance (ANOVA) was used to test the difference between the means of gene expression among the different needle types. Correlation analyses were performed calculating the Kendall rank correlation coefficient.

All calculations were performed using MedCalc version 13 (MedCalc Software, Belgium). A *p* value < 0.05 was considered statistically significant.

## 3. Results

### 3.1. Patients and Tumor Characteristics

Among 1954 EUS performed between November 2018 and October 2019, 37 patients were enrolled (see Supplementary Figure S1) with a mean age of 68.3 years ± 10.9 standard deviation (SD) and with 48.6% being male. Twenty-four (64.9%) lesions were located in the head of the pancreas with a mean diameter at EUS of 32.8 ± 10.6 mm, with 51.4% being borderline resectable and 35.1% diagnosed as locally advanced (Table 1).

### 3.2. RNA Quantification and RIN Evaluation

**Figure 2:**
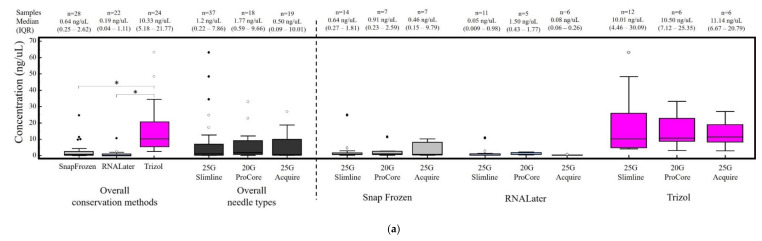

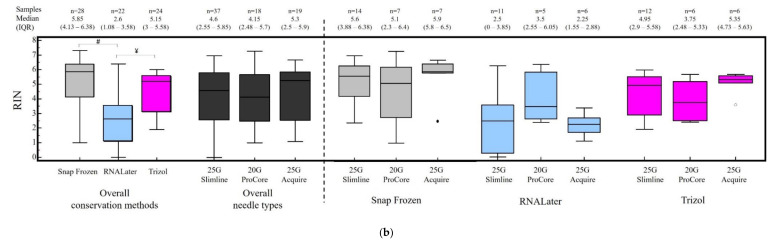
Final RNA concentration (**panel a**) and RNA Integrity Index (RIN) (**panel b**) in relation to the conservation method and needle type. * *p* < 0.0001; # *p* = 0.07 ¥ *p* = 0.08.

Samples conserved in Trizol resulted in a significantly increased median concentration of RNA (10.33 ng/uL) compared with RNALater (0.19 ng/uL; *p* < 0.0001) or snap frozen samples (0.64 ng/uL; *p* < 0.0001) (Figure 2a). No significant difference was observed in terms of RNA quality for Trizol (median RIN 5.15) compared with snap frozen (median RIN 5.85; *p* = 0.8) while both conservation methods resulted in higher quality RNA compared with samples collected in RNALater (2.7; *p* = 0.07 compared with snap frozen; *p* =0.08 compared with Trizol; see Figure 2b). On the other hand, no significant difference in RNA quantity and quality was linked to the needle employed (see Figure 2a,b).

### 3.3. Factors Correlated with RNA Concentration and Integrity Index

**Figure 3:**
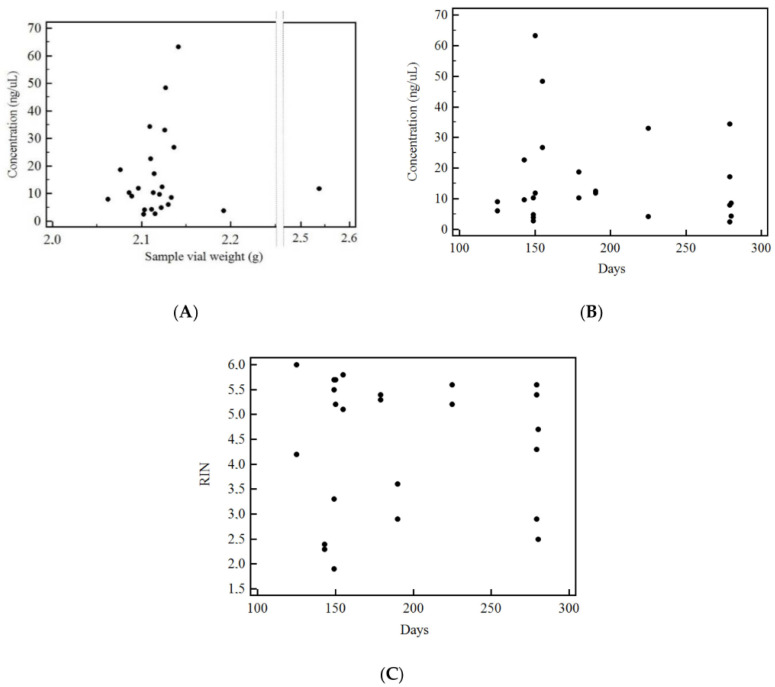
Correlation analyses showing no significance between the final RNA concentration and the weight of the initial sample vial (**A**) (Kendall’s Tau *=* 0.13, *p* = 0.39) or the time elapsed from sampling to RNA extraction (**B**) (Kendall’s Tau *=* 0.03, *p* = 0.84) or RNA integrity and time elapsed from sampling to RNA extraction (**C**) (Kendall’s Tau *=* 0.04, *p* = 0.78).

To determine whether the initial sample (vial) weight or days between tissue sampling and RNA extraction affected the final RNA concentration or quality, correlation analyses were carried out on samples conserved in Trizol. As shown in Figure 3, there was no significant correlation between either weight of the initial sample and RNA concentration (see Figure 3A) or its integrity (Figure 3B) or the time elapsed from sampling to RNA extraction and its integrity (Figure 3C).

### 3.4. Analysis of Contamination of Acinar and Stromal on EUS-Derived PDAC Samples

In order to establish whether one needle was superior to the others in terms of reducing acinar or stromal contamination, real-time PCR (qPCR) analyses were performed for the expression of PRSS1, the gene encoding trypsin as a marker of acinar cells, and of COL1, the gene encoding collagen 1 as a marker of stromal cells.

**Figure 4:**
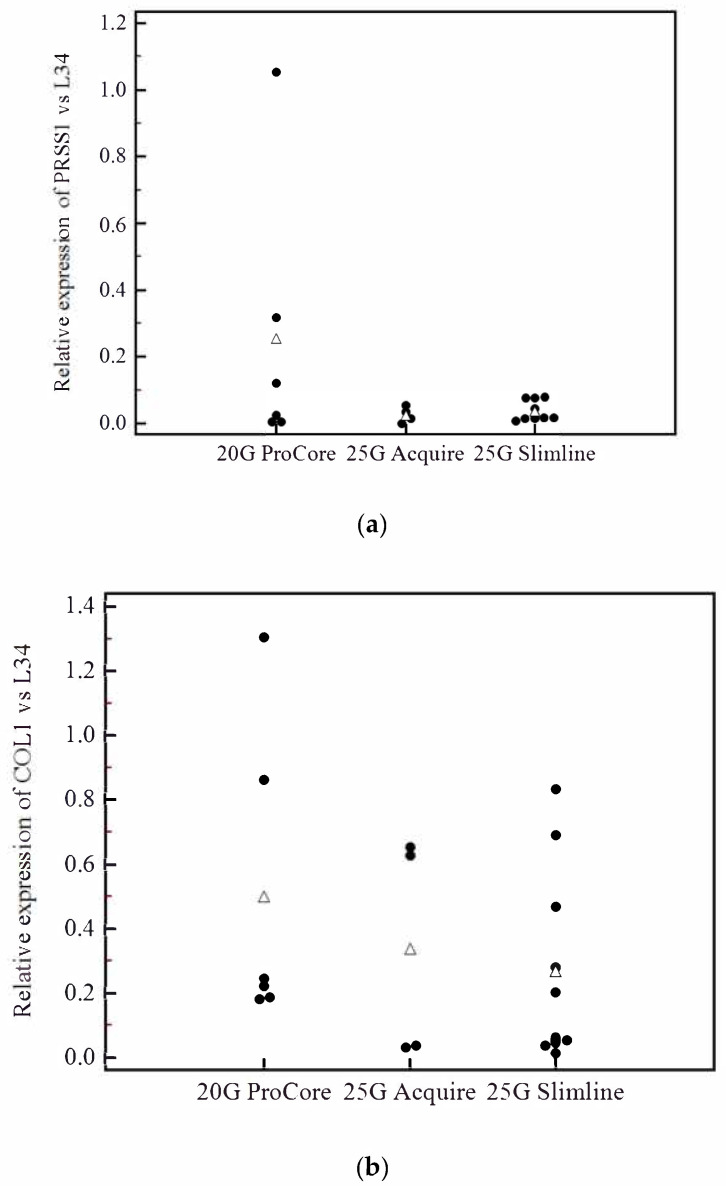
The 20G FNB needle resulted in a higher, despite not significant, mean contamination from acinar and stromal cells based on PRSS1 (**a**) and COL1 (**b**) gene expression.

Out of the total 74 samples collected, 28 were adequate for qPCR considering the thresholds of 60 ng for total RNA quantity and RIN > 3; among these, seven were snap frozen (out of the 28 snap frozen samples = 25%), three were conserved in RNALater (out of the 22 RNALater samples = 13.6%) and 18 in Trizol (out of 24 Trizol samples = 75%). Of the 28 adequate samples, 20 were FNA/FNB matched samples from 10 patients and were therefore employed for qPCR. This analysis indicated that a 20G FNB needle resulted in a higher, despite not significant, mean contamination from acinar (20G ProCore^®^: 0.25, 25G Acquire^®^: 0.04, 25G Slimline^®^: 0.03; *p* = 0.16) and stromal cells (20G ProCore^®^: 0.50, 25G Acquire^®^: 0.33, 25G Slimline^®^: 0.26; *p* = 0.48) (Figure 4).

### 3.5. Evaluation of Prognostic Markers is Feasible on EUS-Derived Samples and May Allow Outcome Prediction

**Figure 5:**
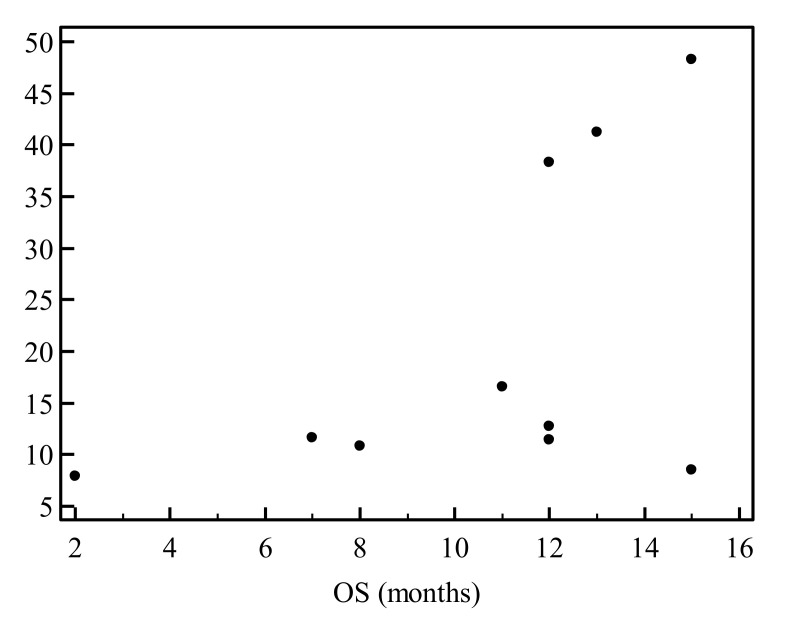
GATA6/ZEB1 ratio shows a trend towards a correlation with overall survival of pancreatic cancer patients (Kendall’s Tau *=* 0.44, *p* = 0.08).

The endodermal transcription factor GATA6 was recently proposed as a marker of the classical PDAC subtype and correlated with better prognosis in patients [8,13,14]. By contrast, the expression of the transcription factor ZEB1, a master regulator of epithelial-to-mesenchymal transition, was associated with poor prognosis and gemcitabine resistance in PDAC [15,16,17]. Gene expression analysis of GATA6 and ZEB1 by qPCR was performed on the 20 selected samples obtained from 10 PDAC patients with both FNA and FNB needles. A significant expression of both genes was detected in 100% of samples (Supplementary Figure S2), indicating the suitability of both needles for gene expression analysis of prognostic PDAC markers. As we hypothesized that the GATA6/ZEB1 ratio may be associated with aggressiveness [13,14,15,16,17], we investigated its correlation (mean value of FNA and FNB needle for each biomarker) with overall survival (OS). Interestingly, the GATA6/ZEB1 ratio showed a trend toward a significant correlation with OS (Kendall correlation coefficient t(tau) = 0.442; *p* = 0.08) (see Figure 5). To confirm these results and highlight the validity of ZEB1, the additional EMT marker SLUG (SNAI2) [28,29,30] was employed and correlated to ZEB1 expression. As reported in Supplementary Figure S3, there was a positive and significant correlation between the two markers (Kendall’s Tau = 0.78; *p* < 0.0001).

### 3.6. Analysis of GAP17 Splicing Variants is Feasible on EUS-Derived Samples

**Figure 6:**
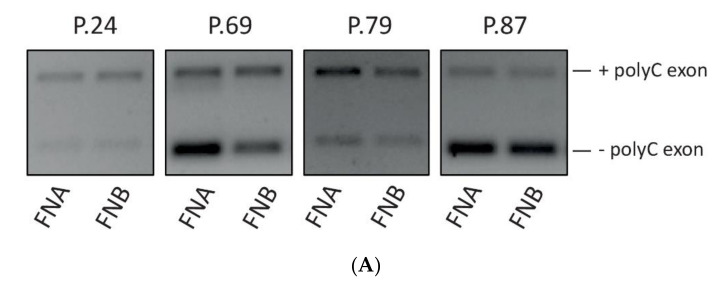

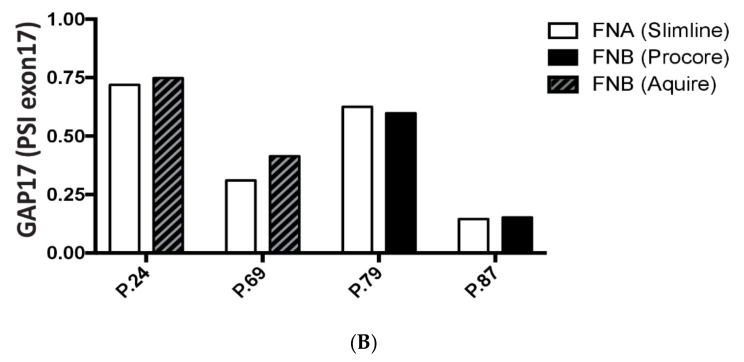
Representative examples of the evaluation of polyC-rich exon inclusion in the GTPase GAP17 (ARHGAP17 gene) in samples obtained from EUS-TA; (panel **A**): RT-PCR with upper and lower bands representing respectively polyC inclusion and respectively; (panel **B**): percentage of Spliced-In Index (PSI/ψ) based on PCR band intensities.

It was recently reported that the recurrent mutations in TP53 lead to widespread changes in alternative splicing in PDAC. In particular, the inclusion of a polyC-rich exon in the GTPase GAP17 (ARHGAP17 gene) was shown to cooperate with a mutant KRAS protein in the activation of the downstream pathway [18]. To determine whether splice variants of the same gene were detectable in samples obtained from EUS-TA, we performed conventional PCR (sq-PCR) analyses using primers in exon that flanked the polyC exon of ARHGAP17. In all 20 samples undergoing sq-PCR we detected the two splice variants of the gene (+the polyC exon) (Figure 6A), regardless of the needle used for sampling. Furthermore, a densitometric evaluation of the percentage of the Spliced-In Index (PSI/ψ) of the polyC exon inclusion (Figure 6B) was feasible in all samples and ranged between 25% and 75%. These results suggest that EUS-FN derived RNA is also suitable for the analysis of splicing events associated with diagnosis. 

## 4. Discussions

Despite the remarkable efforts in the context of translational and clinical research in the past years, the five year survival rate of PDAC patients remains around 8% and has not changed noteworthily in the recent decades [2]. Treatment approaches based on personalized medicine are slowly seeing the light with the acknowledgement by recent guidelines of the importance of the identification of germline mutations and somatic profiling [4,5].

Transcriptional features of tumoral cells have shown great potential for the stratification of patients in several human cancers. In the context of PDAC, two main subtypes have been described on the basis of differential gene expression signatures: the classical and the basal-like. Classical PDAC is characterized by a better prognosis and a response to Folfirinox; the basal-like PDAC is characterized by a worse prognosis and a possible response to gemcitabine-based therapy or targeted therapy [8]. Despite the clusterization that has been possible on the basis of RNA sequencing, there are recent reports of possible discrimination based on single markers [14]. One possible limit of the current classification is due to the fact that the subtyping of PDAC patients has been made only on surgical samples. However, less than 20% of PDAC patients are amenable to surgical resection at diagnosis [12] while the majority of them undergo EUS-TA. Our effort to investigate the best sampling and conservation methods to achieve an adequate quantity and quality of RNA from EUS-TA and to determine its usability for the investigation of prognostic markers aims at overcoming this limit.

RNA extraction from pancreatic tissue is cumbersome and more difficult compared with DNA for the abundance of RNAse in the pancreatic gland that leads to a fast RNA degradation after sample acquisition. Furthermore, PDAC is characterized by a low percentage of tumoral cells for the abundance of stroma and the acquisition through EUS can lead to contamination due to the passage of the needle through the gastric or duodenal wall and through healthy pancreatic tissue [27]. To date, there are a few reports of RNA extraction through EUS-TA with scarce results. In a first study, only 5–10 ng of RNA were extracted from EUS-FNA with no report on the integrity and were used for qPCR [23]. Berry et al. reported to retrieve, with one pass of FNA snap frozen in liquid nitrogen, around 12.9 ug of RNA of relatively low quality (mean RIN of 3) [24]. The extraction of RNA from archival formalin-fixed paraffin-embedded (FFPE) EUS-FNB samples (therefore requiring at least two or three passes) with a 22G Forktip needle has been recently performed [25] but only 28.8% of samples (*n* = 45) resulted in being adequate for nanostring analysis and the RNA quality was not reported. In another study, RNA extraction was performed from 40 snap frozen samples acquired through a standard 22G needle, achieving a high quantity of RNA (1 µg) with the quality assessed by agarose gel electrophoresis and spectrophotometry but not quantitatively reported [26]. These samples were adequate to perform a qPCR analysis of the VEGFR genes. Nevertheless, no study evaluated different conservation methods and needles side-by-side to determine the best methodology for RNA acquisition. Herein, by comparing three needles and three conservation methodologies, we show that the conservation of samples in Trizol is the preferred choice whereas all three needles were equally suitable. In particular, we report that Trizol conservation allows the extraction from one single FNA/FNB pass a median of 300 ng (ranging from 60 ng to 1890 ng) of RNA with a median RIN of 5, making 75% of all Trizol samples adequate to perform qPCR even for delicate analyses such as that of splicing variants. Thus, our results hold promise for good performance in terms of both quantity and quality of RNA for transcriptomics analyses of samples from non-resectable PDAC patients.

Among the three types of needles evaluated (a standard 25G FNA needle, a lateral core-trap 20G FNB needle and a Franseen 25G FNB needle) no single one resulted in a significant superiority in terms of the quantity or quality of RNA retrieved. As for contamination, there was an insignificant higher amount of both acinar and stromal contamination (based on PRSS1 and COL1 gene expression) with the use of the 20G ProCore FNB needle (Figure 4). We can therefore speculate that in the case of studies aiming at evaluating the stromal component, a larger FNB needle might be preferred. Moreover, to provide information suitable for procedure guidelines, we carried out correlation analyses to evaluate whether the initial sample weight or days between sampling and extraction affected the final RNA concentration or RIN. As we observed no significant correlation, our results suggest that relative flexibility can be tolerated in the procedure.

Recently, numerous studies have reported a superiority of the Franseen needle in terms of the adequacy of the tumoral specimen for diagnostic purposes [31,32]. Nevertheless, this superiority might not translate in retrieving a higher quantity or better quality of RNA, as shown from our results. It is true that the 22G Franseen might seem more promising than the 25G one we employed in our study and this has to be further evaluated in prospective studies aiming at molecular analysis. Furthermore, a recent study by Park et al. [22] reported how larger needles retrieve a higher quantity of DNA from EUS-TA. However, possibly due to the differences in terms of stability between DNA and RNA, in our study the 20G needle did not show any benefit compared with other needles.

Recently, the importance of the expression of GATA6 has been highlighted in clinical studies on surgical specimens and in silico. GATA6 is a regulator of pancreatic development and activates epithelial differentiation [13,33]. Notably, GATA6 is highly expressed in classical subtypes and although its evaluation alone was proposed to be sufficient to determine the molecular subtype of PDAC [14], other studies have reported how basal and classical subtypes may coexist [9]. ZEB1 is a transcription factor that promotes tumor invasion and metastatic spread by inducing epithelial-mesenchymal transition and is implicated in gemcitabine-based resistance [15,16,17]. In the present study, the expression of these two prognostic factors was successfully evaluated on all of the 20 samples tested. We found that neither gene was associated with OS in these 10 PDAC patients. However, the ratio between them (GATA6/ZEB1) was much more informative and the correlation with overall survival almost reached a statistical significance in spite of the extremely small cohort analyzed (*n* = 10). This result suggests that combining the evaluation of the expression levels of relatively few genes from EUS-TA derived samples may help predict the prognosis of non-resectable PDAC patients.

Recent studies have clearly shown that splicing is generally deregulated in human cancers including PDAC [34] and that the splicing signature is more suitable to classify patients than the gene expression signature [35]. Thus, we also aimed at exploring the feasibility of detecting prognostic splicing events in our set of samples. A recent paper by Escobar-Hoyos et al. reported that TP53 missense hotspot mutations, which are associated with a more aggressive PDAC, led to the inclusion of polyC exon in the ARHGAP17 gene [18]. As we report that the evaluation of this splicing event is feasible in all of the samples utilized in our study, our preliminary results suggest EUS-derived samples are also suitable for splicing analyses.

The present study has some strengths: it is the first study to specifically evaluate prospectively the combination of three different conservation methods and of three different needles for the acquisition of RNA from PDAC samples; the RNA retrieved was of high quantity and good quality and was successfully used to perform qPCR to determine contamination from acinar and stromal cells, to evaluate the expression of prognostic markers such as GATA6 and ZEB1 and also to determine splicing events. The patients were from similar stages and the prognosis of all patients was prospectively recorded for a correlation with biomarkers.

The study carries the intrinsic limitations of a pilot one, which aimed at testing experimental conditions and feasibility such as the relatively small sample, the lack of a head-to-head comparison of all three needles and conservation methods as this was not ethically acceptable and the execution of qPCR only on a set of patients. The PRSS1 and COL1 gene expression evaluation on EUS-acquired normal pancreatic tissue for comparison was also not carried out, as this was not ethically acceptable. Moreover, the ratio between GATA6/ZEB1 is only speculative and not validated as also the overall survival was partial and not definite. Lastly, the mutational status of P53 was not investigated in the patients undergoing the GAP17 splice event evaluation.

In conclusion, in our study EUS-acquired samples of PDAC conserved in Trizol resulted in a significantly higher concentration and sufficient integrity of RNA and hence usable to perform qPCR for tissue and prognostic biomarkers including the evaluation of splicing events. Further studies on a larger set of patients and of RNA sequencing on EUS-acquired samples are needed in order to evaluate its feasibility and investigate additional prognostic markers, valid also for locally advanced diseases.

## Figures and Tables

**Table 1:** Patient and tumor characteristics.

	Total Patients Enrolled (*n* = 37)
Age, years (mean ± SD)	68.3 (± 10.9)
Sex, male, n (%)	18 (48.6%)
Location of the lesion	
Head	24 (64.9%)
Body-tail	13 (35.1%)
Resectable	4 (10.8%)
Borderline resectable	19 (51.4%)
Locally advanced	13 (35.1%)
Metastatic	1 (2.7%)
Dimension of lesion at EUS, mm (mean ± SD)	32.8 (± 10.6)

SD: Standard Deviation.

## Supplementary Materials

The following are available online at https://www.mdpi.com/2073-4409/9/12/2561/s1, Figure S1: Flow chart of patient selection for RNA extraction from EUS-acquired pancreatic cancer tissue. Figure S2: GATA6 and ZEB1 vs. L34 expression evaluation was feasible in 20 samples. Figure S3: ZEB1 and SLUG(SNAI2) show a positive significant correlation (Kendall’s Tau = 0.78; *p* < 0.0001). Table S1: List of Primers employed for gene expression and splicing variant evaluation.
