# Supplementary material for: RNA Extraction from Endoscopic Ultrasound-Acquired Tissue of Pancreatic Cancer Is Feasible and Allows Investigation of Molecular Features
Source: Cells. 2020 Nov 30;9(12):2561. doi: 10.3390/cells9122561 (PMC7761443; doi:10.3390/cells9122561)
Supplement: Supplementary file 1 [file cells-09-02561-s001.pdf]

Supplementary Materials: The following are available online at www.mdpi.com/xxx/s1, Figure S1: Flow chart of patient selection for RNA extraction from EUS-acquired pancreatic cancer tissue. Figure S2: GATA6 and ZEB1 vs. L34 expression evaluation was feasible in 20 samples. Figure S3: ZEB1 and SLUG(SNAI2) show a positive significant correlation (Kendall's Tau = 0.78; $p < 0.0001$). Table S1: List of Primers employed for gene expression and splicing variant evaluation.

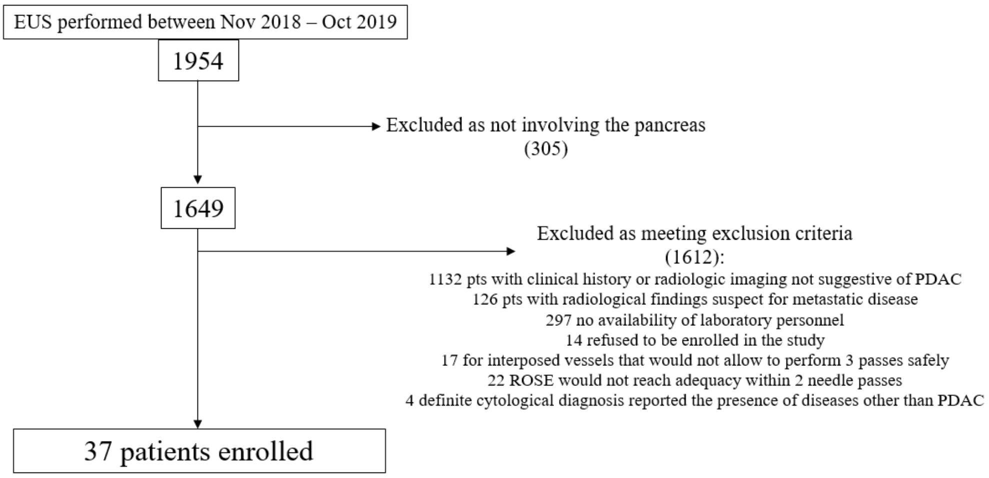

Figure S1. Flow chart of patient selection for RNA extraction from EUS-acquired pancreatic cancer tissue.

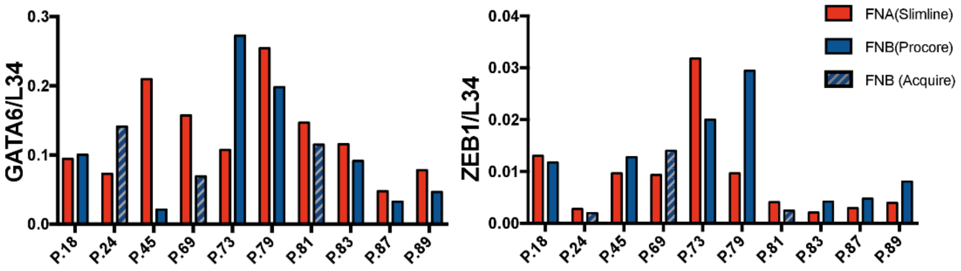

Figure S2. GATA6 and ZEB1 vs. L34 expression evaluation was feasible in 20 samples.

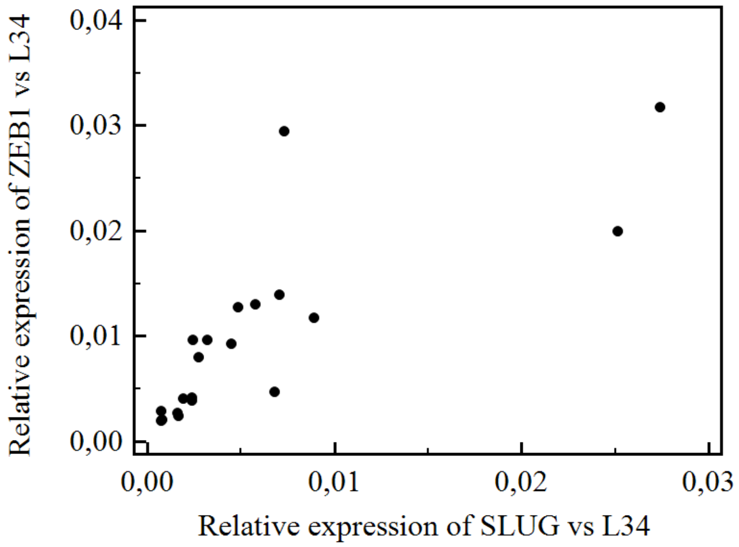

Figure S3. ZEB1 and SLUG(SNAI2) show a positive significant correlation (Kendall's Tau = 0.78; $p < 0.0001$).

Table S1. List of Primers employed for gene expression and splicing variant evaluation.

Gene	Forward	Reverse
ARHGAP17	CTCACCACCCCGAGTTCTAA	TGTTGTTTCTCCCTGGTGCT
Col1a1	CAACAGCCGCTTCACCTACA	TCAATCACTGTCTTGCCCCA
GATA6	GCCAACTGTCACACCACAAC	CATAGCAAGTGGTCTGGGC
L34	GGCCCTGCTGACATGTTTCTT	GTCCCGAACCCCTGGTAATAGA
PRSS1-3	TGAAGCCTCCTACCCTGGAA	TGTCCATTGCAGACCACAGG
ZEB1	ACTCAACTACGGTCAGCCCT	TGGGCGGTGTAGAATCAGAG
SLUG	CAAGGCGTTTTCCAGACCCTG	TTGACCTGTCTGCAAATGCTCT
